# Feeding strategies and intraspecific competition in German yellowjacket (*Vespula germanica*)

**DOI:** 10.1371/journal.pone.0206301

**Published:** 2018-10-26

**Authors:** Michelina Pusceddu, Alessandra Mura, Ignazio Floris, Alberto Satta

**Affiliations:** Dipartimento di Agraria, Sezione di Patologia Vegetale ed Entomologia, Università di Sassari, Sassari, Italy; Ben-Gurion University of the Negev, ISRAEL

## Abstract

The German yellowjacket (*Vespula germanica*) is an opportunist predator and a scavenger, whose eclectic diet also includes honey, brood, dead and live honey-bees. There is no evidence in this species of coordinated attacks against bees involving other conspecifics, although intraspecific competition has been already reported between two or more individuals during feeding. Our aim was to gain further knowledge on the feeding behavior of *V*. *germanica* in order to evaluate its role in an apiary. Sight observations of predation and necrophagy behaviors were carried out at the ground level near hives. We also investigated how intraspecific competition can influence the feeding display in this species. Our results confirm the major role of the German yellowjacket as a scavenger, because its diet is based mostly on bee carrions. Intraspecific competition during feeding was sometimes observed. When these events occurred, the interference of another wasp led to the bee escaping only in three cases. Our study also revealed that intraspecific competition events increase when the resource is fresh (predation *vs* necrophagy), and that the number of competing wasps was significantly higher when the food consisted of pupae and drones, compared to adult bees. When competition involved two individuals (the most frequent case), the winner was frequently the first wasp to reach the resource in both predation and necrophagy events. This suggests that the energy invested in foraging or predating activity and in defence of prey is usually rewarded.

## Introduction

*Vespula germanica* is a social wasp that is widespread in much of the world. Its native distribution includes Europe, northern Africa, northern India, Korea and China [1], however it is also a successful invasive species in Argentina [2] and Australia [3]. The global success of this species is mainly determined by its food plasticity, which makes it easily adaptable to different environments [4,5,6]. In fact, the European wasp is an opportunist predator and a scavenger because its eclectic diet includes small prey, vertebrate and invertebrate carrions, food and garbage from humans, and also carbohydrates from nectar, fruits and honeydew [7,8,9,10]. Coelho and Hoagland [11] reported that in the late summer and early autumn, German yellowjackets feed on dead honey-bees found at the ground level near hives. They also steal honey or prey on adult bees and brood [12,13]. Usually the wasps dismember their prey at the cervix and/or petiole level [14], and fly off with selected parts of the dead bee [15]. Aebi and Aebi [16] reported that *Vespula* spp. prefer to sequestrate the abdomen, while according to Duncan [15] and Winston [17] they prefer the thorax, and according to Free [12], they prefer the thorax and abdomen over the head, but specific studies on *V*. *germanica* are lacking.

Despite this opportunistic feeding behavior, German yellowjacket foragers preserve the memory of their past experience, in fact they return to eat at previously successful foraging sites [9,6,18,19] and show an associative learning between local landmarks (visual, spatial and odor cues) and a certain food source [9,5,20,21,22,23]. The foraging behavior of *V*. *germanica* may change in relation to the habitat: wasps returned to the original feeding site more frequently in closed habitats than in open ones, probably because closed habitats offer more landmarks to guide the foragers to the food source [2].

Other abiotic factors that affect the daily foraging activity of *V*. *germanica* include rain, low and high temperatures and low light (e.g. fog) which reduce foraging [24]. Social mechanisms may also contribute to successful finding of alimentary resources. In fact, *V*. *germanica* foragers are attracted by the presence of conspecifics at food sources by local enhancement processes [25,26] and they can influence other naive foragers to search for an odor sampled inside the nest [27]. Lozada et al. [10] show that there is evidence of social communication for forager recruitment even at a distance from the resource: when foragers return to the nest after the foraging trip, the subsequent number of wasp foragers was approximately four times higher compared to when communication with the nest was not possible.

However, individual foraging and independent hunting typology have been described for this species that explain how several individuals from different colonies can find themselves at the same foraging site and defend their own prey from other conspecifics [12,13]. Intraspecific competition for food as an aggressive interaction between two or more German yellowjackets has also been reported by Parrish and Fowler [28] also when food supplies were not scarce. In areas with a high wasp density but without enough prey, cannibalism has also been observed [3].

In areas where it has been introduced, *V*. *germanica*, may also have an ecological impact on the natural ecosystem and is considered a pest for certain human activities [29,30,31,32]. In particular, the economic damage caused by this wasp on beekeeping is due to the costs incurred for destroyed or damaged hives (approx. 9% of the total number of hives) and to productivity losses [29], due also to the strong competition for the honeydew resource [33]. Controlling the *V*. *germanica* population, which is normally based on the use of poison-baits [29,34], also has a financial cost.

Although these studies show that yellowjacket represents a problem in many countries, the biology of this species in native areas has not been studied sufficiently. In a previous work [13] we studied the agonistic interactions between *V*. *germanica* and *A*. *mellifera* [13], our aim in the present work was to assess the economic damage of this species for the beekeeping industry through the evaluation of the impact on the bee colonies of the wasp's feeding activity. Through behavioral observations in the field, we investigated the predation and necrophagy behavior, as well as the dismemberment pattern and preferences in the collection of body parts. Finally, the influence of intraspecific competition on the feeding display of this wasp was also assessed.

## Materials and methods

### Experimental apiary

The study was performed in an experimental apiary in northwest Sardinia in fall 2017, at the experimental farm of the University of Sassari, Department of Agriculture (latitude 40°46’23”, longitude 8°29’34”). The apiary, which is located in an area where the predation activity of *V*. *germanica* on honeybees has been reported since September 2014 [13], comprised 10 *A*. *mellifera ligustica* colonies maintained in Dadan-Blatt hives containing 10 combs each. During the experimental period, the hives were checked every week to confirm the presence of the queen, as well as the pollen and nectar provision, and to evaluate the sanitary status (symptoms of diseases and varroosis) [35].

The presence of *V*. *germanica* in our experimental apiary was also monitored from August 2017 using two wasp-traps (a bottle with beer) placed near the hives. After the arrival of the wasps (29/09/2017), the weekly number of *V*. *germanica* individuals along a transect (0.70 m x 4.25 m) traced in line with the apiary was recorded.

### Behavioral observations

The feeding behavior of wasps on bees at the ground level was observed using the “all-occurrences sampling” method [36], by which the frequencies of a series of behavioral events were recorded as set out in the ethogram described below. We focused on the attacks targeted on *isolated bees* and *bees removing other bees* at the ground level (failed attacks and predation), and on dead bees (necrophagy behavior). It was not possible to record blind data because our study involved animals in the field. A total of 99 observation hours were conducted during the period in which the predatory and foraging activity of *V*. *germanica* is more intense (from late September 2017 to early November 2017) [12]. Two operators simultaneously observed the ground surface under five hives in two sessions per day, each lasting 45 min. These observations were conducted by sight, and the frequency (number of events per unit of time) for each of the observed predation and necrophagy behaviors was annotated. In addition, for each predation and necrophagy events, the degree of dismemberment and the specific body part sequestered by the wasps were reported.

### Intraspecific competition between wasps

For each predation or necrophagy event in which intraspecific competition between wasps was observed, the arrival of subsequent wasps after the first was registered as well as the type of resource for which they competed (adult bees, drones, or pupae). When possible, the winner (the wasp that monopolized the resource) was identified in terms of arrival order (first, second etc.).

### Ethogram at the ground level

***Attack*–**The wasp grasps the bee and starts biting it (usually on the abdomen or between the head and thorax) [13].

***Predation****–*The wasp kills the honeybee. The wasp usually goes on to dismember and consume the honeybee, or to carry off parts to its offspring (see below) [37].

***Necrophagy***–The wasp consumes the body parts of dead bees that it finds at the ground level [11].

***Dismemberment*–**After predation or during necrophagy, the wasp divides the honeybee into different parts (head, thorax, abdomen; head + thorax and abdomen; head and thorax + abdomen) [12]. If the resource is a dead bee not intact (e.g. without head or without abdomen), the wasp may divide it further (e.g. head and thorax; thorax and abdomen).

***Sequestration****–*After predation or necrophagy and having divided the honeybee into different parts, the wasp flies off with one of the parts, usually the thorax [15,17]. In some cases, the wasp may also carry the whole bee.

***Retreat***–The wasp escapes when the attack has not been successful and the honeybee defends itself effectively [13].

***Killing wasp****–*Wasps can be killed by a single bee sting or by balling [13].

***Intraspecific competition*–**Aggressive interactions between two or more individuals of *V*. *germanica* during feeding [28].

### Statistical analysis

A chi squared test was used to measure the proportional difference in intraspecific competition, between necrophagy and predation events. The same test was used to measure the proportional difference in dismemberment and in sequestration behavior between competitive and non-competitive events during predation and also during necrophagy. To reduce the chances of a type I error, continuity correction was used for the chi-squared test when the sample size was less than 200 [38]. The Wilcoxon rank sum test (unpaired comparisons) was used to compare the number of wasps that compete in the predation and necrophagy events. To reduce the chances of a type I error in this analysis, we used Bonferroni correction in the case of multiple testing with significance set at α = 0.05/2 = 0.025.

The Kruskal Wallis test was used to compare the number of wasps that competed (in the predation + necrophagy events) for different resources: pupae, drones and adult bees. Subsequently Dunn’s post-hoc test with Bonferroni correction was used to find the significant differences. We also verified the correlation between the density of wasps under 10 hives and the number of competitive events detected in the following three hours using non-parametric Spearman correlation. All tests were carried out using R v 3.0.2 implemented with library: exactRanktests, coin and asbio.

Raw experimental data are available in supporting materials (S1 Datafile).

## Results

### Attack behavior

**Fig 1:**
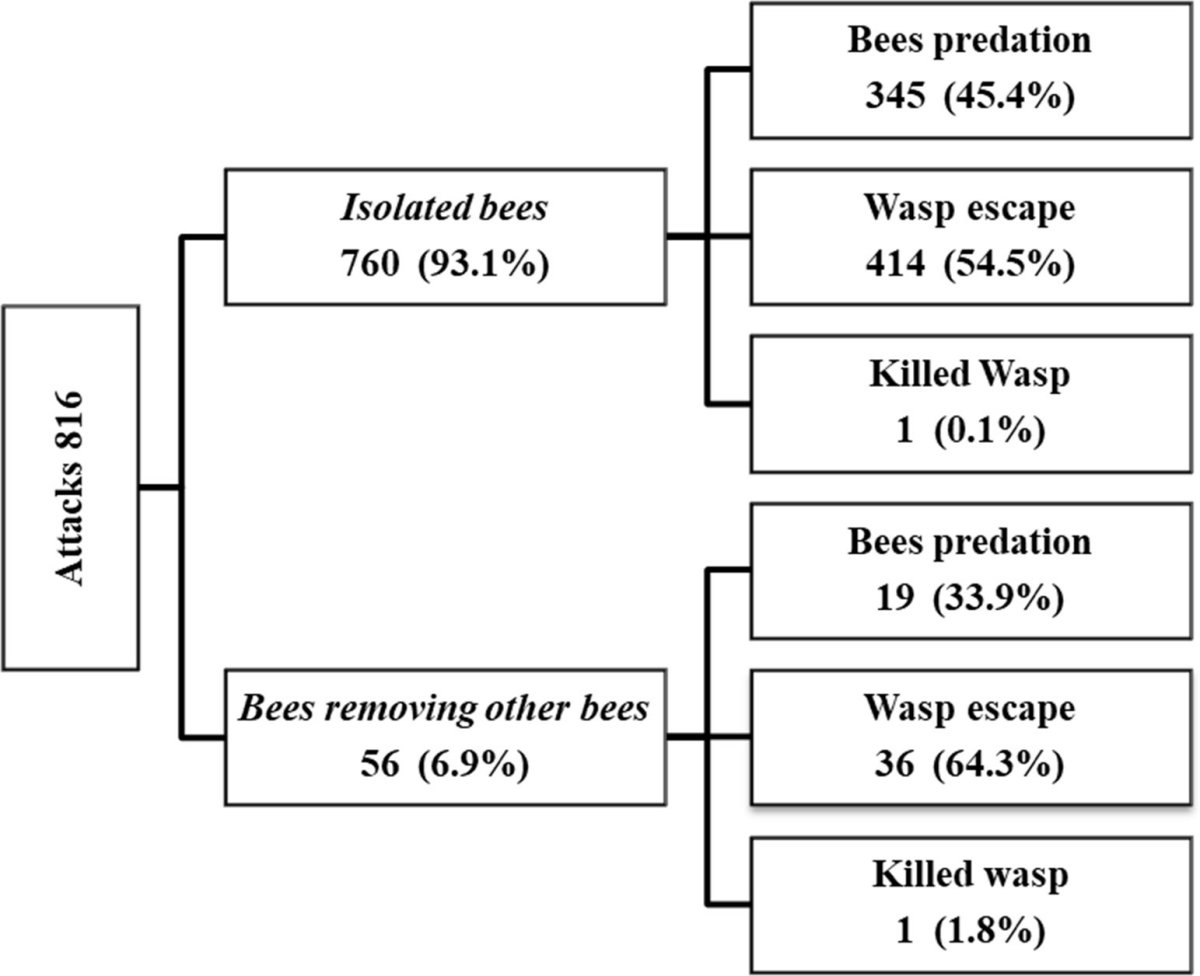
Outcome of attacks conducted at ground level toward *isolated bees* and *bees removing other bees*.

We observed 816 attacks at ground level in 99 hours, representing ~ 8.2 attacks per hour. Specifically, 760 attacks (93%) were targeted at *isolated bees* and 56 (7%) at *bees removing other bees*. The most frequent outcome was the wasp escaping without the prey (450 events, corresponding to 55%), while predation occurred on another 364 occasions (45%). Only in two cases was the wasp killed (0.3%). No significant differences in the success rate of the attacks comparing the two targets: *isolated bees* and *bees removing other bees* were observed (45% vs 34%; *chi-squared* = 2.33, *df* = 1, *P* = 0.1269). The attack data are summarized in Fig 1.

### Predation behavior

**Fig 2:**
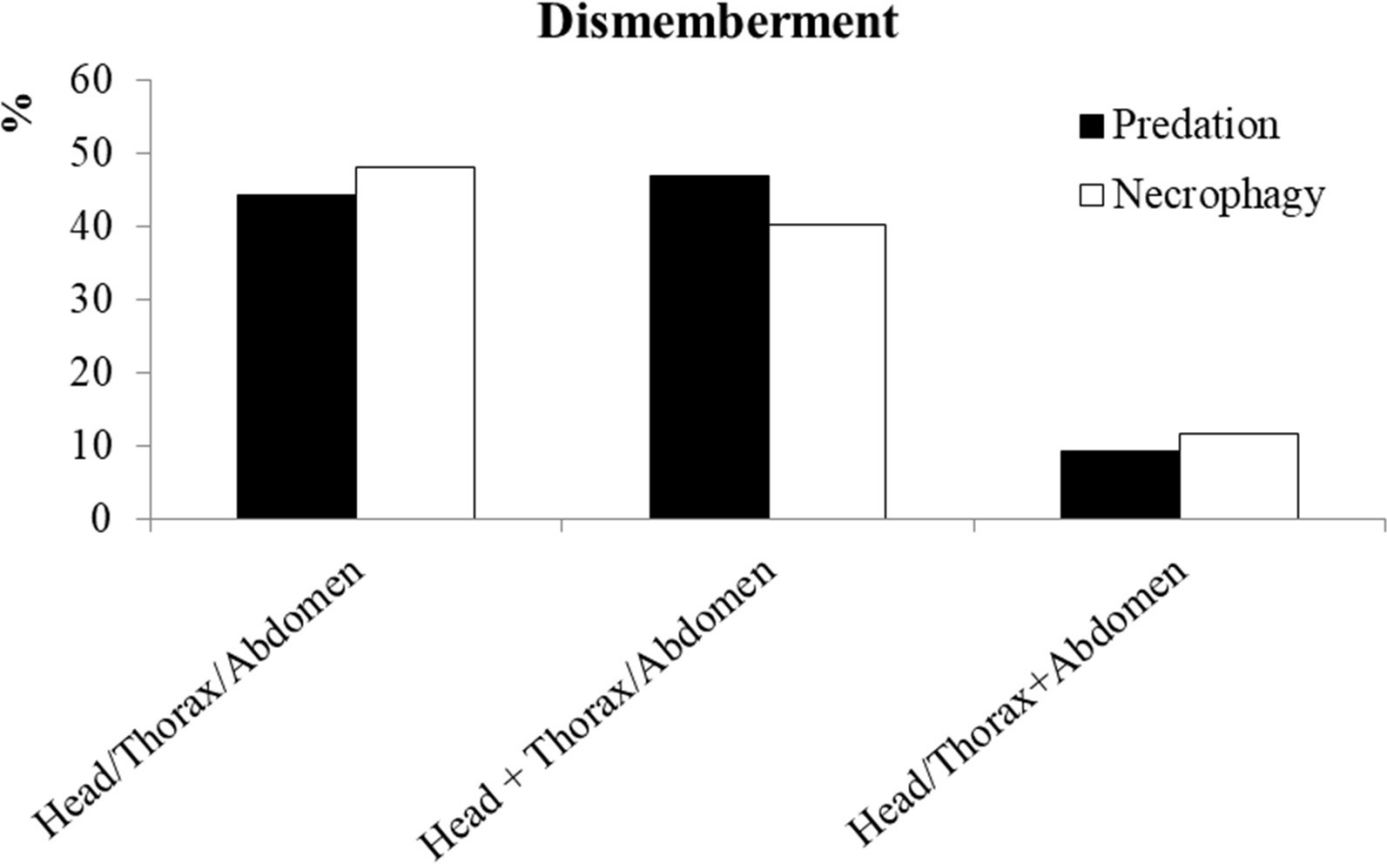
Proportion of different types of dismemberment in predation (n = 304) and necrophagy (n = 241) events.

**Fig 3:**
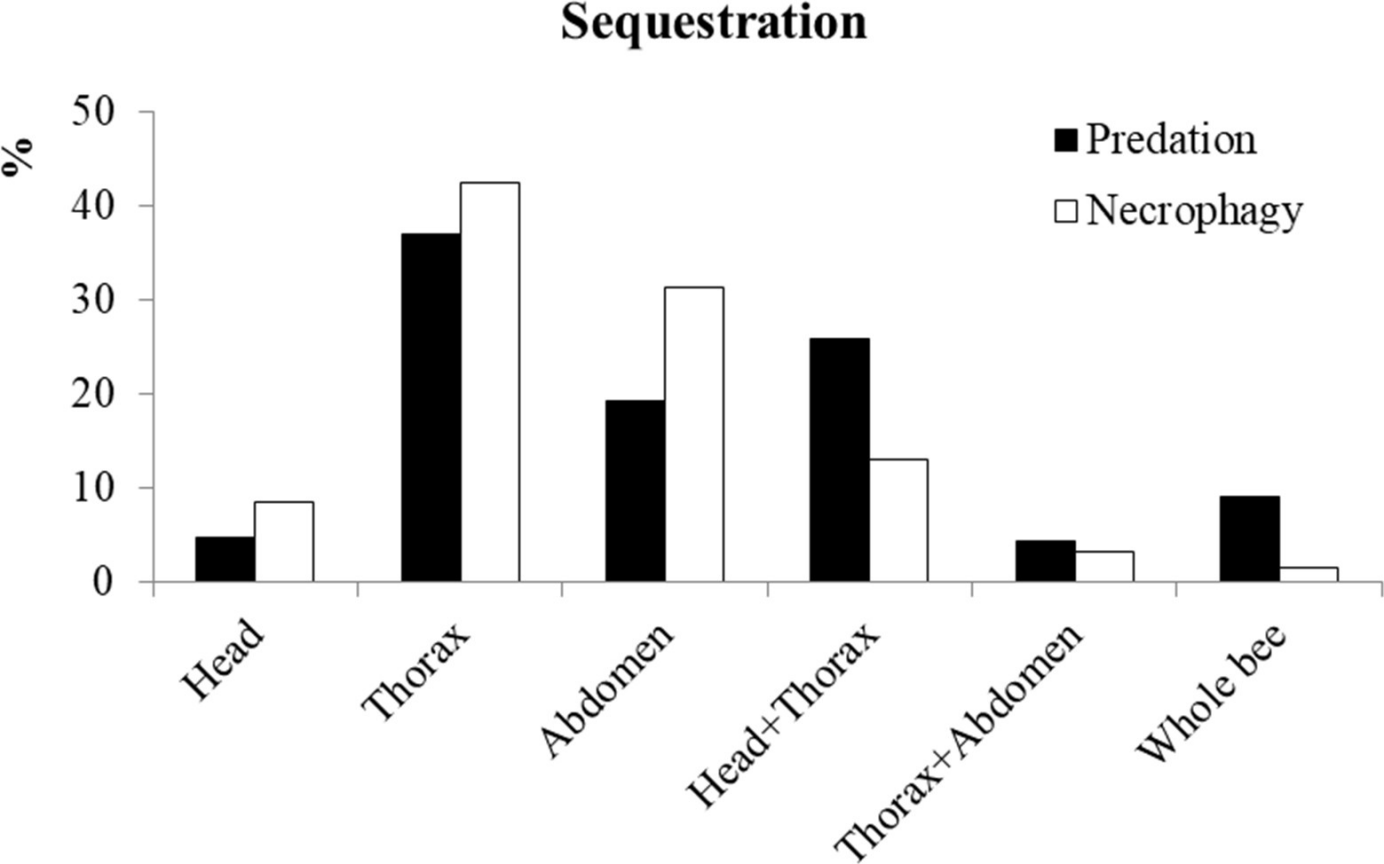
Proportion of different bee parts sequestered in predation (n = 322) and necrophagy (n = 575) events.

Of the 364 bee predation events observed, 304 cases (83%) also involved victim dismemberment (Fig 2). The wasp eating its prey on-site was observed 42 times (11%), but more often, after predation and dismemberment, sequestration occurred (322 events, corresponding to 88%) with a preference for the thorax followed by the abdomen and the head + thorax (Fig 3). In 29 cases (9%) the prey was sequestered without dismemberment.

### Necrophagy behavior

We observed 775 bee necrophagy events at ground level in 99 hours, corresponding to ~ 7.8 cases per hour. Necrophagy on *integral carrions* was observed 707 times (91%), while, in the remaining cases, we observed the wasp eating *non-integral carrions* (Head+Thorax or Thorax+Abdomen). The pattern of dismemberment observed on *integral carrions* was similar to that observed during predation (Fig 2), however the frequency was significantly lower (34% vs 83%) (chi-squared = 234.90, df = 1, P < 0.001). Conversely, when the target of necrophagy was *non-integral carrions*, dismemberment was always reported. After necrophagy, sequestration was observed in 575 cases, corresponding to 74%. This frequency was statistically lower than in the case of predation (88%) (chi-squared = 30.13, df = 1, P < 0.001). In the other 200 occasions (26%), the carrion was consumed on-site. The preference of body parts sequestered is summarized in Fig 3. In nine cases (1.6%) the carrions were sequestered without dismemberment.

### Intraspecific competition

We observed intraspecific competition in both predation and necrophagy events however, in the first case, the rate at which it occurred was significantly higher (50% vs 23%) (*chi-squared* = 77.34, *df* = 1, *P* < 0.001). Only in three cases (0.7%) did the interference of another wasp lead to the failure of the attack and the bee escaping.

The average number of competing wasps was significantly higher when food consisted of pupae (4.8 ± 1.1) and drones (4.2 ± 0.5) compared to adult bees (2.3 ± 0.1) (Kruskal Wallis *chi-squared* = 58.33, *df* = 2, *P* < 0.001; Dunn’s post-hoc test with the Bonferroni correction: pupae-drones = 1; pupae-adult bees = 1e-06; drones-adult bees = 0).

A positive linear correlation between the wasp density in the observation area and the number of competition events was also recorded (S = 10.5928, P = 0.0269, rho = 0.81).

In addition, when competition involved two individuals, frequently the winner was the wasp that first reached the resource in both predation (64%) and necrophagy (66%) events.

**Fig 4:**
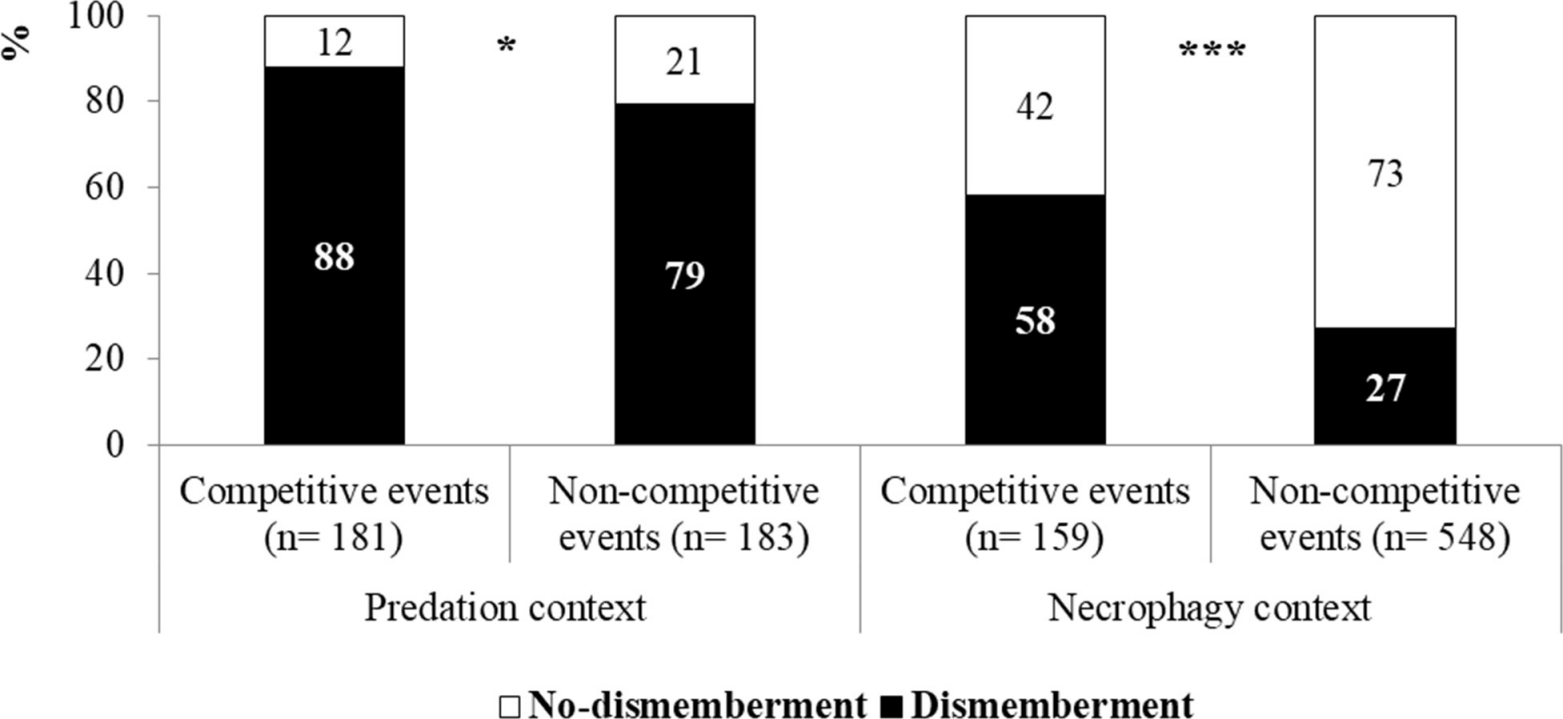
Percentage incidence of the dismemberment activity in competitive and non-competitive events in a predation (n = 181 and n = 183, respectively) and in a necrophagy (n = 159 and n = 548, respectively) context. In the necrophagy context only the events on *integral-carrions* were considered.

**Fig 5:**
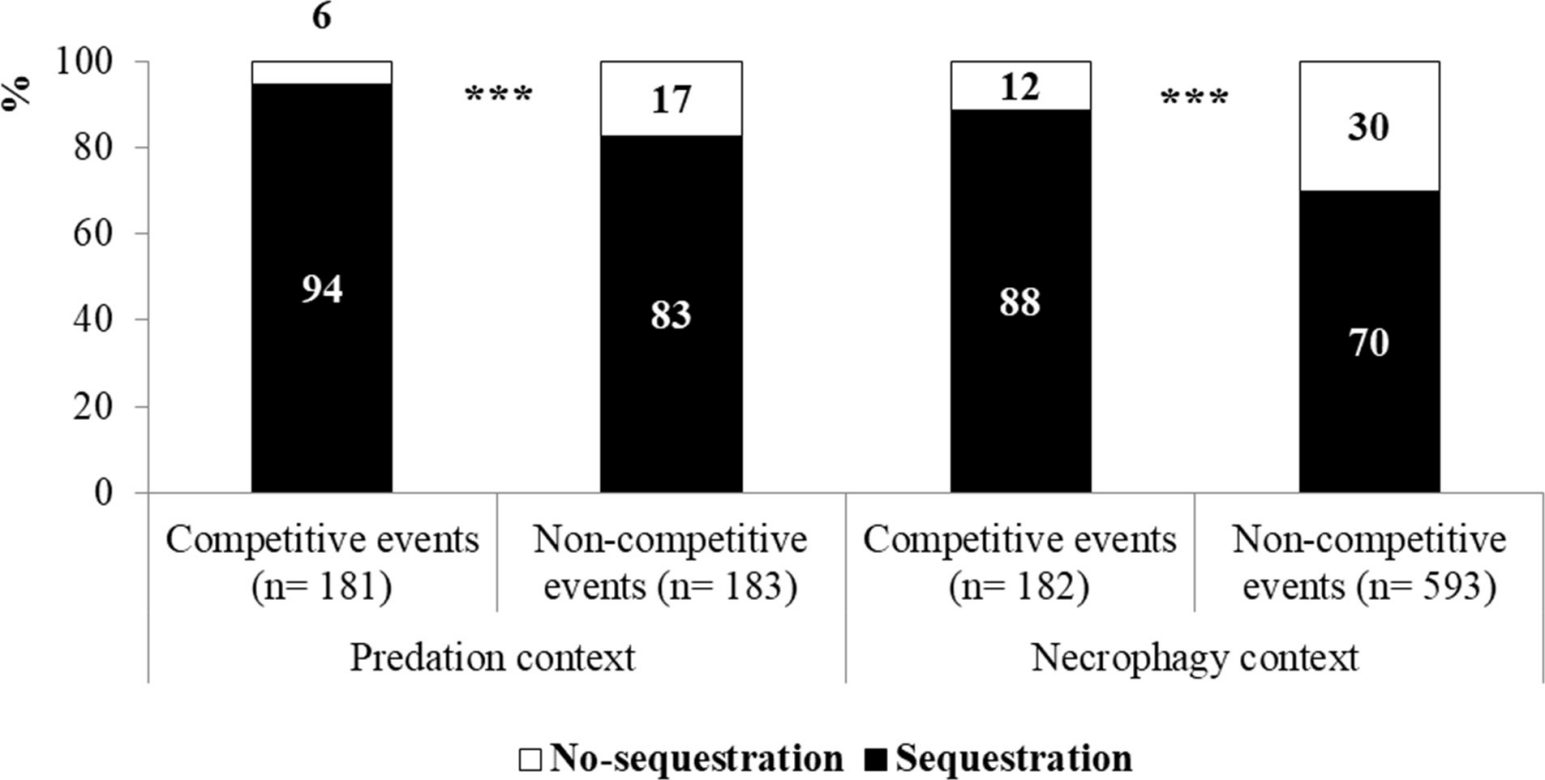
Percentage incidence of the sequestration activity in competitive and non-competitive events in a predation (n = 181 and n = 183, respectively) and in a necrophagy context (n = 182 and n = 593, respectively).

Finally, the influence of competition on dismemberment and sequestration behaviors was also observed. The frequency of dismemberment significantly increased when competition occurred, both in predation and necrophagy events as shown in Fig 4 (Predation: chi-squared = 4.29, df = 1, P = 0.038; Necrophagy: chi-squared = 50.24, df = 1, P < 0.001). Similar results were also observed for sequestration (Predation: *chi-squared* = 11.61, *df* = 1, *P* < 0.001; Necrophagy: *chi-squared* = 24.32, *df* = 1, *P* < 0.001 Fig 5).

## Discussion

In a previous study regarding the agonistic interactions between *A*. *mellifera* and *V*. *germanica*, we observed that the wasp attacked the hive entrance infrequently due to the low success rate of this strategy, while preferring a specialized attack aimed at weak or isolated adult honeybees at ground level [13]. The results of the present work showed that the number of attacks on live bees at the ground level was balanced by the number of necrophagy acts. Considering that only half of the attacks on live bees were successful with predation, we can affirm that the diet of *V*. *germanica* is based mostly on bee carrions. This suggests that in our apiary context the main role of the German yellowjacket was as a scavenger and highlights the highly opportunistic behavior of the wasp which, avoiding any physical fight with the live bees defending themselves from the attack, minimizes the risk of dying or losing prey and obtains a good reward from carrions. The evolution of this low-cost foraging strategy can be explained by the optimal foraging theory, which in particular postulates a trade-off between the foraging behaviour and lifetime fitness [39]. However, considering the large plasticity of the alimentary behaviour of this species [4,5,6], there may be a serious impact on honey bee colonies when favourable environmental conditions occur (wasp nest density, food source availability, weak colonies). However, contrary to the general opinion of beekeepers, our research did not find evidence that the presence of the *V*. *germanica* represents a threat for the hives in native areas.

Unlike findings described for *Vespa tropica* [40], *Vespa velutina* and *Vespa crabro* [41], we never observed *V*. *germanica* capturing foragers in flight returning to the hive, despite being easy prey because they are weighed down by nectar or pollen load, and “tired” after the flight activity. *V*. *germanica* probably does not adopt this attack strategy because it is smaller than the other species of wasps cited above, preferring to attack its prey on the ground. Another attack strategy that we observed at the ground level was aimed at *bees removing other bees*. In this case the attacked bee is less ready to defend itself as it is engaged in another activity. In addition, it is weighed down by the carried bee who, if still alive, fights to avoid its removal from the nest. However, when this attack strategy is successful, the reward that the wasp obtains is represented by the removed bee and not by the bee that is doing the removing.

During the period of observation (from late September 2017 to end of October 2017) and within individual observation sessions, we noted a progressive increase in the number of foraging wasps at the same site. This can be explained by considering that German yellowjacket foragers are attracted by the presence of conspecifics at food sources [25,26] and by the fact that they preserve the memory of their past experience, particularly regarding the reward obtained at a food site [9,6,18,19]. Although this type of social facilitation probably took place during our experiments, competitive interactions between wasps were also observed. It is highly probable that several individuals from different colonies may find themselves at the same foraging site and defend their own prey from other conspecifics [12]. On the other hand, competitive interactions between individuals of *V*. *germanica* during feeding, even when the food resource was not scarce, have been described by Parrish and Fowler [28].

As in other animal species, a greater density of individuals at the same site favors the intraspecific competition for resources [42,3,43]. In fact, our data showed a positive correlation between the number of wasps present at the ground level and the number of agonistic interactions for food. However, only in three cases (0.7%) of the total intraspecific competition events observed, did the interference of another wasp lead to the failure of the previous attack and the bee escaping.

Our study also revealed that intraspecific competition increases when the resource is fresh (predation *vs* necrophagy). This outcome suggests that necrophagy, compared to predation, may represent the best trade-off between reward and energy costs (in terms of risk, energy investment in foraging on carrion and also in defending food from conspecifics).

We found the highest number of competing wasps when the food consisted of pupae and drones, compared to adult worker bees. In fact, pupae and drones are a larger and "unarmed" source of food compared to workers, and consequently represent a low risk for the wasp. Furthermore, Free [12] reported that the alimentary preference of *V*. *germanica* and *V*. *vulgaris* for pupae compared to adult bees, is probably due to the difference in cuticle hardness.

In addition, intraspecific competition led to a significant increase in the rate of the dismemberment and sequestration in both predation and necrophagy events. However, it did not affect the dismemberment pattern and body parts sequestered by the wasp, which was preferentially the thorax, probably due to its higher protein content compared to the head and abdomen [12].

Our study also revealed that many German yellowjacket individuals are able to sequester a whole bee. It has been already reported that *V*. *germanica* have a higher load-lifting capacity, compared to *V*. *squamosa* and *V*. *maculifrons* [11]. The highest theoretical load can be calculated considering different factors, including for example flight muscle mass. However, the wide intraspecific variation in load-carrying capacity, mainly depending on the size of the wasp, can influence the foraging ability of each individual [11]. Individual size and arriving early at the resource are factors that can also play an important role in intraspecific competition [44]. In fact, we found that when competition involved two individuals, the winner was frequently the first wasp that reached the resource in both predation and necrophagy events. This suggests that the energy invested in foraging or predating and in defence of their own prey is usually rewarded.

Finally, in the future it would be interesting to quantify how the individual size influences intraspecific competition in this species, particularly in relation to the competition involving two individuals which was the most frequent event observed in our study.

## Supporting information

S1 Datafile (XLSX)
